# Prevalence and Comorbidities Among Individuals With Rheumatoid Arthritis in the Saudi Arabian Context

**DOI:** 10.7759/cureus.53992

**Published:** 2024-02-10

**Authors:** Mushabab Alghamdi, Mansour Y Somaily, Shahenda Alemam, Saeed Majadah, Abduaelah Ali H Hassan, Abdullah A Meshary, Saad Ahmad A Alasmri, Munif S Ali, Abdullah A Alsubaie, Elhadi Miskeen

**Affiliations:** 1 Department of Internal Medicine and Rheumatology, College of Medicine, University of Bisha, Bisha, SAU; 2 Department of Medicine, Rheumatology Division, Armed Forces Hospital - Southern Region, Khamis Mushait, SAU; 3 Department of Medicine, Rheumatology Division, Khamis Mushait General Hospital, Khamis Mushait, SAU; 4 College of Medicine, King Khalid University, Abha, SAU; 5 Department of Obstetrics and Gynecology, College of Medicine, University of Bisha, Bisha, SAU

**Keywords:** saudi arabia, ra patients, profile, prevalence, comorbidities, rheumatoid arthritis

## Abstract

Background: Rheumatoid arthritis (RA) in Saudi Arabia (SA) is a significant health concern with a notable impact on individuals and the healthcare system. This study aimed to investigate the prevalence and profile of comorbidities in patients with RA.

Methodology: This is a retrospective descriptive study involving 150 RA patients from August 2022 to August 2023, which was conducted at Khamis Mushait General Hospital, a major healthcare institution in SA. We examined the medical records to gather pertinent information. Stata Statistical Software: Release 18 (2023; StataCorp LLC, College Station, Texas, United States) was used for data analysis. The examination focused on sociodemographic factors, disease duration, prescribed medications (including methotrexate and biologic therapy), and the presence of comorbidities. Approval for the study was obtained from the Institutional Review Board of the Aseer Ministry of Health (approval number: H-06-B-091).

Results: The study found a high prevalence of comorbidities in patients with RA. Around 96.7% of the patients had at least one documented comorbidity, highlighting this population's burden of additional health conditions. The most common comorbidity observed was anemia, affecting 48.7% of the patients. Other frequently observed comorbidities include hypertension, hyperlipidemia, diabetes mellitus, osteoporosis, interstitial lung disease, chronic renal disease, stroke, and coronary artery disease. The factors influencing comorbidities included an odds ratio of 1.086 (p=0.025), while being male was associated with lower odds (odds ratio=0.529, p=0.017). Additionally, disease duration (odds ratio=1.164, p=0.007), methotrexate use (odds ratio=2.553, p=0.001), and receiving biologic therapy (odds ratio=3.488, p<0.001) were significant contributors to comorbidities.

Conclusion: These findings highlight the need for comprehensive approaches to address RA and its associated comorbidities. Research and awareness initiatives are essential to understand better the specific nuances of RA in SA, leading to improved diagnostic and treatment strategies for the needs of the local population.

## Introduction

Rheumatoid arthritis (RA) is a persistent autoimmune condition marked by extensive inflammation, primarily emphasizing joint involvement [1]. The worldwide prevalence of RA is estimated to be approximately 1% worldwide [2]. RA is associated with substantial morbidity, leading to joint deformity, functional impairment, and reduced quality of life in affected individuals [3]. However, beyond common manifestations, RA is a complex systemic disorder with various comorbidities [4].

Comorbidities are additional medical conditions that coexist with the primary disease and can significantly affect the overall health outcomes of patients. Understanding the prevalence and patterns of comorbidities in patients with RA is crucial for optimizing clinical management, predicting disease outcomes, and implementing comprehensive care strategies [5,6].

Recognizing comorbid conditions in RA is vital because of their potential influence on disease outcomes and patient prognosis [7]. Comorbidities may develop due to shared risk factors, due to common pathogenic mechanisms, or as an outcome of prolonged chronic inflammation and its systemic repercussions [8]. They have the potential to impact diverse organ systems, leading to elevated morbidity, mortality, and healthcare utilization among individuals with RA [9].

Comorbid conditions are increasingly recognized to play a critical role in treating RA [10]. Comorbidities can influence disease activity, treatment response, functional impairment, and quality of life in patients [11]. Cardiovascular diseases, metabolic syndrome, respiratory comorbidities, osteoporosis, and mental health disorders are commonly observed as comorbid conditions in RA [12]. A comprehensive approach that addresses the joint manifestations of RA and its associated comorbidities is crucial for optimizing patient care and improving long-term outcomes [13,14].

These examples highlight the wide range of comorbid conditions in RA and their significant impact on disease outcomes and patient management [15]. Healthcare providers must recognize and address these comorbidities in the holistic management of RA [16,17].

Like many other countries, Saudi Arabia (SA) faces challenges posed by RA and its associated comorbidities [18]. In SA, the high burden of RA and its comorbidities has significant implications for the healthcare system [19]. However, research on the comorbidities observed in patients with RA within the Saudi Arabian population is limited.

Therefore, in this study, we aimed to address this knowledge gap by reviewing the medical records of patients with RA from Khamis Mushait General Hospital, a major healthcare institution in SA. By examining a sample of 150 patients, from the period August 2022 to August 2023, we sought to explore the prevalence and patterns of comorbidities among this population, providing insights into the burden of additional medical conditions experienced by patients with RA in SA.

## Materials and methods

Study design

This retrospective descriptive investigation included 150 patients diagnosed with RA from August 2022 to August 2023.

Study area

The study was conducted at Khamis Mushait General Hospital, a prominent healthcare institution in SA. In the Aseer region, Khamis Mushait is a referral center for patients with rheumatic diseases, including RA.

Study population

The study population comprised individuals diagnosed with RA, confirmed through adherence to hospital guidelines that encompassed both clinical evaluation and laboratory analysis.

Clinical Assessment

This involved assessing for symmetric joint involvement, morning stiffness lasting at least 30 minutes, presence of joint swelling and tenderness, as well as systemic symptoms such as fatigue and weight loss.

Laboratory Testing

Diagnostic procedures included measuring elevated levels of inflammatory markers such as C-reactive protein and erythrocyte sedimentation rate, identification of rheumatoid factor, and employing imaging modalities such as X-rays to evaluate joint inflammation and damage.

Data collection

Relevant information, including sociodemographic details, disease duration, comorbidities, and treatment data for RA (such as prescribed disease-modifying antirheumatic drugs (DMARDs) and biologic therapies), was collected by examining the medical records.

Data analysis

This study used descriptive statistics to examine and present the prevalence and patterns of comorbidities among patients with RA. Categorical variables, including comorbidities, were analyzed by calculating the frequencies and percentages. In contrast, continuous variables, such as age and disease duration, were summarized using diverse measures, including means, standard deviations, medians, and interquartile ranges. The choice of the measure depends on the distribution characteristics of the data. Logistic regression analysis was conducted to estimate the likelihood of comorbidities detection. The dataset underwent cleaning and preparation, ensuring the inclusion of variables such as comorbidities (coded as binary: 0 for absent and 1 for present) and patient characteristics (e.g., age, sex, disease duration).

## Results

The research involved 150 individuals diagnosed with RA. The majority of the study participants were females, constituting 128 (85.3%). Individuals aged 18-49 accounted for 70 (46.7%), and 108 (72%) were married. Additionally, 62 (41.3%) had a body mass index (BMI) within the normal range (18.5-24.9), 98 (65.3%) resided in urban areas, and 103 (68.7%) were employed. The average duration of RA among the participants was 8.7 years. These findings collectively present a comprehensive profile of the demographics and health characteristics of the individuals included in the study (Table 1).

**Table 1:** Characteristics of the studied population (n=150)

Variables		Number	Percentage
Gender	Male	22	14.7
	Female	128	85.3
Age (in years)	18-49	70	46.7
	50-64	56	37.3
	65-74	19	12.7
	≥75	5	3.3
Marital status	Single	7	4.7
	Married	108	72
	Divorced	1	0.7
	Widow	34	22.7
Body mass index	18.5-24.9	62	41.3
	25-29.9	42	28
	30-34.9	29	19.3
	35-39.9	13	8.7
	≥40	4	2.7
Residency	Urban	98	65.3
	Rural	52	34.7
Work status	Employed	103	68.7
	Not employed	47	31.3
Years of diagnosis	8.7 (min 1, max 20)		

**Figure 1:**
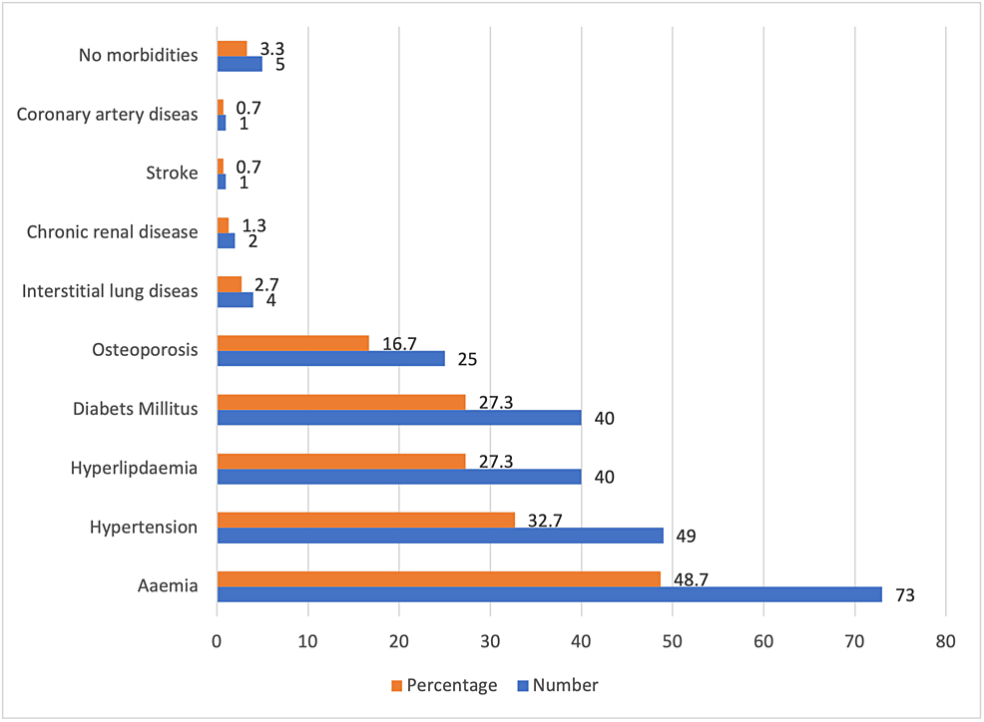
Profile of comorbidities among RA patients RA: rheumatoid arthritis

The findings indicated that 145 patients (96.7%) had at least one documented comorbidity, with 50 patients (33.3%) exhibiting two or more comorbidities. Among the documented comorbidities, anemia was the most prevalent at 73 cases (48.7%), followed by hypertension at 49 cases (32.7%), hyperlipidemia at 40 cases (27.3%), diabetes mellitus at 40 cases (27.3%), osteoporosis at 25 cases (16.7%), interstitial lung disease at four cases (2.7%), chronic renal disease at two cases (1.3%), stroke at one case (0.67%), and coronary artery disease at one case (0.67%) (Figure 1).

Among the prescribed DMARDs, the most commonly utilized drugs were methotrexate, sulfasalazine, leflunomide, and hydroxychloroquine. Approximately 37 patients (24.7%) were prescribed two or more DMARDs. Biologic therapy was administered to 55 patients (36.7%), with adalimumab being the most frequently prescribed first-line biologic therapy for 40 patients (27%). In cases of secondary loss of response, 24 patients (16%) received second-line biologic therapy, such as tocilizumab (9%) or abatacept (7%).

Logistic regression analysis examined the association between comorbidities and various patient factors in patients with RA. Table 2 overviews the logistic regression results, including the coefficients, odds ratios, 95% confidence intervals, and p-values. This interpretation highlights the significant associations between certain patient factors (age, sex, disease duration, methotrexate use, and biologic therapy) and the likelihood of comorbidities among RA patients. The non-significant associations (BMI, residence, work status, and marital status) suggest that these variables may not strongly predict comorbidities in this context.

**Table 2:** Logistic regression results of the comorbidities profile among RA patients RA: rheumatoid arthritis; BMI: body mass index

Variable	Coefficient	Odds ratio	95% CI	p-value
Age (in years)	0.082	1.086	1.010-1.167	0.025
Gender (male)	-0.637	0.529	0.312-0.896	0.017
Disease duration	0.152	1.164	1.041-1.302	0.007
Methotrexate use	0.937	2.553	1.453-4.488	0.001
Biologic therapy	1.249	3.488	2.028-6.006	<0.001
BMI	0.031	1.031	0.975-1.090	0.326
Residency (urban)	0.489	1.631	0.958-2.777	0.072
Work status	0.201	1.222	0.715-2.090	0.470
Marital status	-0.076	0.927	0.588-1.461	0.722

For each year increase in age, the odds of having comorbidities among patients with RA increased by 8.6% (odds ratio=1.086, p=0.025). This association is statistically significant, suggesting that advanced age increases the probability of comorbidities. Lower odds were observed among males than females (odds ratio=0.529, p=0.017). This relationship was statistically significant, suggesting that being male is protective against comorbidities in patients with RA. With each additional year of disease duration, the odds of comorbidities increased by 16.4% (odds ratio=1.164, p=0.007).

Patients with RA using methotrexate had 2.6 times higher odds of comorbidities than those not using methotrexate (odds ratio=2.553, p=0.001). This relationship is statistically significant, suggesting that methotrexate use is associated with an increased likelihood of comorbidities. In addition, RA patients receiving biologic therapy had 3.5 times higher odds of comorbidities than those not receiving biologic therapy (odds ratio=3.488, p<0.001). 

Residing in an urban area was associated with a higher likelihood of comorbidities; however, this relationship was not statistically significant (odds ratio=1.631, p=0.072). Additionally, work status was not significantly associated with comorbidities among patients with RA (odds ratio=1.222, p=0.470). This finding suggests that work status may not significantly predict comorbidities in this population. Moreover, marital status was not associated with comorbidities in patients with RA because the coefficient was not statistically significant (odds ratio=0.927, p=0.722).

## Discussion

The study population consisted of 128 females (85.3%) and 22 males (14.7%), reflecting the higher prevalence of RA in women, which is commonly observed worldwide [20]. The finding that the male sex is protective against comorbidities in patients with RA aligns with the literature that has reported a higher prevalence of comorbidities in female patients with RA [21,22]. Hormonal and genetic factors and differences in healthcare-seeking behavior may contribute to this sex disparity in comorbidity burden among patients with RA.

The common age group was 18-49 representing 46.7%. The average disease duration among the participants was 8.71 years, with a standard deviation of 3.1 years, indicating a population with substantial chronicity of RA. This allowed us to examine how comorbidities vary in prevalence, severity, and management needs among younger and older individuals with RA. This is important because the duration of RA can influence disease progression, treatment response, and likelihood of developing comorbidities [23]. A longer disease duration is often associated with more severe joint damage, higher levels of inflammation, and an increased risk of comorbidity [24]. The positive association between age and comorbidities among RA patients is consistent with previous studies' findings. Numerous studies have shown that older age is a significant risk factor for developing and accumulating comorbid conditions in RA [25,26]. The progressive nature of RA and prolonged exposure to inflammation over time may contribute to an increased likelihood of comorbidities in older patients. Hence, studying a population with a substantial duration of RA enables a more comprehensive understanding of the cumulative effects of the disease and its associated comorbidities.

The substantial occurrence of comorbid conditions among RA patients, as unveiled in this study, underscores the requirement for holistic management approaches that tackle not only the underlying disease but also the accompanying comorbidities. The finding that 96.7% of patients with RA in the cohort had at least one documented comorbidity aligns with previous research emphasizing the high comorbidity burden in this population [27,28]. 

The finding that with each additional year of disease duration, the odds of having comorbidities among RA patients increased by 16.4% is notable and suggests a significant association between disease chronicity and comorbidity burden. This finding aligns with prior research that has consistently shown an augmented risk of comorbidities in patients with RA as disease duration increases [29,30].

Aggressive treatment strategies aimed at achieving disease control, such as early initiation of DMARDs and biologic therapies, when indicated, have been shown to improve outcomes and potentially reduce the burden of comorbidities [31,32]. It is worth noting that while disease duration is an essential factor, the development of comorbidities in RA is influenced by multiple complex interactions, including genetic predisposition, lifestyle factors, and other comorbidity-specific risk factors. Therefore, a comprehensive approach that considers these factors is necessary to understand and manage the comorbidity burden in patients fully.

The most commonly prescribed DMARDs were methotrexate, sulfasalazine, leflunomide, and hydroxychloroquine. These medications control RA activity and reduce joint damage [33]. It's worth noting that approximately 24.7% of the patients were prescribed two or more DMARDs, suggesting the potential need for combination therapy in cases of inadequate response or increased disease activity. It indicates a potential need for combination therapy in cases of inadequate response or increased disease activity. 

The finding that patients with RA using methotrexate have 2.6 times higher odds of comorbidities than those not using methotrexate is significant. This raises important concerns regarding the potential impact of this medication on the development of comorbid conditions. Although methotrexate is an effective DMARD commonly prescribed for RA, its association with comorbidities has been a subject of interest and discussion in the literature.

Several factors may contribute to the observed association between methotrexate use and the increased odds of comorbidities. First, methotrexate suppresses the immune system, which may increase susceptibility to infections and other immune-related comorbidities [34,35]. Second, long-term methotrexate use has been associated with potential adverse effects, including liver toxicity, bone marrow suppression, and gastrointestinal disturbances, which could contribute to the development or exacerbation of comorbidities [36-38]. It is important to note that the relationship between methotrexate and comorbidities is complex and the benefits of medication in controlling RA disease activity often outweigh potential risks. It is essential to recognize that the observed association between methotrexate use and comorbidities does not imply causation. Further research is needed to investigate this relationship, considering potential confounding factors, such as disease severity, treatment duration, and concomitant medications. Long-term prospective studies and systematic reviews are essential to provide a more comprehensive understanding of the impact of methotrexate on the development of comorbidities in patients with RA.

Biologic therapy, a class of medications specifically targeting inflammatory molecules involved in RA, was prescribed to 36.7% of patients. Among the biological agents, adalimumab was the most frequently prescribed first-line natural agent (27%). However, a subset of patients (16%) required second-line biologic therapy because of a secondary loss of response to the initial treatment. Tocilizumab and abatacept were the most commonly used second-line biologics in this study. The association between biologic therapy and comorbidities underscores the importance of closely monitoring RA patients receiving these treatments. Regular assessments of safety parameters, including infection screening and surveillance for malignancies, are essential to ensure the appropriate use of biologic therapy and mitigate potential risks [39]. Additionally, patient education shared decision-making and involving patients in treatment decisions [40].

The significant association between biologic therapy and comorbidities in patients with RA was consistent with previous studies highlighting an increased risk of infections and malignancies associated with specific biological agents [41,42]. Biologic therapy is highly effective in controlling RA inflammation; however, the potential for adverse events and comorbidity development should be carefully considered when prescribing these agents.

The non-significant associations between BMI, residency, work status, marital status, and comorbidities among patients with RA suggest that these factors may not substantially influence comorbidity risk in this population. However, it is important to interpret these findings cautiously and recognize that many factors, including genetic predisposition, lifestyle choices, and socioeconomic factors, influence the comorbidity development in RA. Regarding BMI, while obesity has been associated with increased inflammation and disease severity in RA, its direct impact on comorbidity risk remains uncertain and conflicting in the literature [43,44]. Future research should incorporate more comprehensive evaluations of comorbidities, expand sample sizes, and employ longitudinal study designs to enhance our understanding of the influence of these factors on comorbidity risk in patients with RA. Furthermore, delving into alternative potential factors like socioeconomic status, lifestyle choices, and psychosocial elements might offer valuable insights into the intricate connection between these variables and the risk of comorbidities in RA. While methotrexate remains a cornerstone therapy for RA, its associations with comorbidities and disease manifestations are multifaceted and not fully elucidated. The apparent inconsistencies in the literature underscore the need for further research to better understand the complex interplay between methotrexate, RA, and associated comorbidities. Additionally, personalized treatment approaches and careful monitoring of patients are essential to optimize therapeutic benefits while minimizing potential risks and adverse effects.

While the current study established a significant association between age and comorbidities, it is worth noting that comorbidity development is a complex and multifactorial process. Other factors, such as disease duration, lifestyle factors, genetic predisposition, and treatment regimens, may also contribute to the comorbidity burden in patients with RA. Future research should consider these additional factors to provide a more comprehensive understanding of the interplay between age, disease characteristics, and comorbidity.

Limitations

This study has some limitations to be considered. The study included 150 RA patients, and while this sample size provides valuable insights, it might limit the statistical power for detecting associations, particularly when examining less prevalent comorbidities or exploring subgroups within the population. Despite efforts to extract comprehensive information from medical records, there might be missing or incomplete data, affecting the accuracy and comprehensiveness of the findings. The study design (cross-sectional) constrains the ability to establish causation or discern the chronological association between RA and comorbidities.

## Conclusions

This study provides valuable insights into the prevalence and patterns of comorbidities among patients with RA at Khamis Mushait General Hospital in SA. The high prevalence of comorbidities highlights the importance of comprehensive care and underscores the need for a multidisciplinary approach to RA management. The findings of this study contribute to the understanding of RA-related comorbidities in the Saudi Arabian population and serve as a basis for further research and clinical interventions aimed at improving the outcomes of patients with RA in the region.
